# Current Evidence to Propose Different Food Supplements for Weight Loss: A Comprehensive Review

**DOI:** 10.3390/nu12092873

**Published:** 2020-09-20

**Authors:** Mikiko Watanabe, Renata Risi, Davide Masi, Alessandra Caputi, Angela Balena, Giovanni Rossini, Dario Tuccinardi, Stefania Mariani, Sabrina Basciani, Silvia Manfrini, Lucio Gnessi, Carla Lubrano

**Affiliations:** 1 Department of Experimental Medicine, Section of Medical Pathophysiology, Food Science and Endocrinology, Sapienza University of Rome, 00161 Rome, Italy; mikiko.watanabe@uniroma1.it (M.W.); davide.masi@uniroma1.it (D.M.); Alessandra.caputi@uniroma1.it (A.C.); angela.balena@uniroma1.it (A.B.); s.mariani@uniroma1.it (S.M.); sabrinabasciani@yahoo.it (S.B.); lucio.gnessi@uniroma1.it (L.G.); carla.lubrano@uniroma1.it (C.L.); 2 Department of Endocrinology and Diabetes, University Campus Bio-Medico of Rome, 00128 Rome, Italy; giovanni.rossini@unicampus.it (G.R.); d.tuccinardi@unicampus.it (D.T.); s.manfrini@unicampus.it (S.M.)

**Keywords:** insulin resistance, metabolic syndrome, obesity, weight loss, botanicals, dietary supplements, nutraceuticals

## Abstract

The use of food supplements for weight loss purposes has rapidly gained popularity as the prevalence of obesity increases. Navigating through the vast, often low quality, literature available is challenging, as is providing informed advice to those asking for it. Herein, we provide a comprehensive literature revision focusing on most currently marketed dietary supplements claimed to favor weight loss, classifying them by their purported mechanism of action. We conclude by proposing a combination of supplements most supported by current evidence, that leverages all mechanisms of action possibly leading to a synergistic effect and greater weight loss in the foreseen absence of adverse events. Further studies will be needed to confirm the weight loss and metabolic improvement that may be obtained through the use of the proposed combination.

## 1. Introduction

The prevalence of obesity has been rising steadily for the past decades all over the world [1,2] leading to an increase in prevalence of many complications of weight excess, some of which are well acknowledged, such as Type 2 Diabetes (T2D), obstructive sleep apnea syndrome (OSAS), non-alcoholic fatty liver disease (NAFLD), and cardiovascular disease [3,4,5,6], while others are emerging and currently being investigated [7,8].

Several strategies have been proposed for the treatment of weight excess and its detrimental consequences, ranging from dietary regimens [9,10,11,12,13,14,15], to pharmacological treatments [16,17], physical exercise [18], and psychological approaches [19]. Most of these are safe [16,20,21], although some have risen concern [22,23]. However, despite leading to improvement in many cases, the major issue is the presence of adverse events and reduced compliance. So-called super foods and food supplements have gained much popularity in recent years, for the generalized perception that natural substances may be synonym of health and balance. Despite this being partially false, given the many adverse events that may derive from natural compounds ingestion, it is indeed true that many of the currently commercialized dietary supplements are virtually devoid of major side effects. 

Currently available food supplements feature several purported mechanisms of action, such as improvement of carbohydrate metabolism, increased lipolysis or energy expenditure, and reduced hunger. A vast amount of literature is available, often of low quality, making navigation hard, and adequate recommendation to those who ask for advice remain very challenging. It is also crucial to keep dosage in consideration as it is acknowledged in many fields that dosing can make the difference between beneficial effects and toxicity, and too often commercially available supplements provide highly variable amounts without even properly acknowledging it on labels. Lately, the Italian Ministry of Health has put out an alert recommending that all botanicals should undergo testing similar to actual drugs, and many countries will surely follow. Appropriate evaluation of available evidence is therefore crucial to start with. So, herein, we aimed at summarizing available evidence regarding supplements most likely to be effective in aiding weight loss, classifying them by their purported mechanism of action. We conclude that higher quality evidence is much needed, but many of the investigated products seem to be effective, although of little clinical relevance when taken alone. Therefore, combinations aiming at targeting more than one mechanism of action should be adequately studied both from an efficacy and safety point of view in order to assess synergistic or additive actions in the absence of major adverse events. 

## 2. Methods

Literature was reviewed up until March 2020 to investigate the efficacy and safety of dietary supplements towards weight loss. The research was conducted on MEDLINE, Cochrane Library, EMBASE, and Web of Science databases by using the following keywords: “name of investigated food supplement” and “obesity” or “weight loss”. Studies meeting the following criteria were included: (1) case-control studies, cohort studies, observational prospective and retrospective studies, randomized clinical trials (RCTs); meta-analyses (2) reported body weight or body mass index (BMI) measurements over time; (3) female or male only, or both genders enrolled; (4) sufficient detail reported about dietary supplement studied; (5) studies written in English.

Six independent reviewers (RR, AC, AB, DM, GR) evaluated the title, abstract, and keywords of selected papers, and full articles were retrieved if deemed appropriate. References of retrieved articles, reviews, and meta analyses on the topic were assessed for additional studies. All reports were evaluated by RR, AC, AB, DM, and GR for inclusion, and two reviewers resolved eventual disagreements (MW and DT). Data extraction included year of publication, country where the study was conducted, patient gender, mean age, mean BMI, inclusion and exclusion criteria, sample size, study design, intervention, eventual co-intervention, efficacy (body weight, BMI change), and safety outcomes. The authors of the included studies were contacted for missing values where required. Studies whose supplement dosing could not be obtained neither through study protocol nor manuscript nor direct contact with investigator were excluded. 

For each supplement, six independent operators (RR, DM, AB, AC, GR) performed an assessment of the quality of the supporting evidence, based on the GRADE (Grading of Recommendations Assessment, Development and Evaluation) criteria [24]. The quality of evidence for the specific dietary supplement was defined as:

- High if based on one or more updated, high-quality systematic reviews based on at least two high quality primary studies with consistent results;
- Moderate, if based on one or more updated systematic reviews of high or moderate quality based on at least one high-quality primary study or two primary studies of moderate quality with consistent results;
- Low, if based on a limited number of clinical studies or one or more systematic reviews of variable quality based on primary studies of moderate quality with inconsistent results.

A recommendation for every food supplement was then provided based on the quality of evidence, the strength of preclinical evidence, the clinical relevance, and the safety. Taking into consideration such recommendations, a combination of supplements leveraging all mechanisms of action was then suggested.

## 3. Results

Twenty-one dietary supplements were included in the study and classified based on their primary mechanism of action in: food supplements with a primary impact on nutrient absorption (Table 1); food supplements with a primary impact on appetite regulation (Table 2); food supplements with a primary impact on energy expenditure regulation (Table 3); food supplements with a primary impact on fat metabolism (Table 4); food supplements with a primary impact on carbohydrate absorption (Table 5). None of the selected dietary supplements were considered to be supported by high-quality evidence, eight of them (green tea, white kidney bean, caffeine, bitter orange, diacylglycerol, resveratrol, grapefruit, chromium) were considered to be supported by moderate-quality evidence, while the other thirteen were considered of low-quality evidence. In addition, the range of dosages commonly adopted in different studies, the mechanisms of action, and the side effects were reported for each food supplement.

### 3.1. Reduced Nutrients Absorption as Purported Mechanisms of Action

Commercially available obesity medication orlistat exerts its weight lowering effect through the intestinal inhibition of fat absorption. Many food supplements exert their beneficial action through the same pathway, where lipids or carbohydrates absorption is delayed or limited to some extent. Notably, most of these compounds also recognize other mechanisms of action possibly contributing to their beneficial effect on metabolism, such as gastric emptying delay influencing appetite (Table 1).

#### 3.1.1. Green Tea

Green tea (GT) is an unfermented, popular beverage made from the leaves of the plant *Camellia sinensis,* historically used for medicinal purposes and, in recent decades, studied for its potentially beneficial health effects. Catechins, such as epigallocatechin-3-gallate (EGCG), and caffeine (CAF), the predominant component of tea, have been confirmed to possess a broad range of biological activities, such as body weight reduction, metabolic syndrome (MetS) improvement, cardiovascular diseases (CVDs) and cancer prevention, and protection against neurodegeneration [25]. Further, green tea is proven to be safe, with few inconsistent side effects or adverse events [26,27].

One of the most effective ways GT can contrast obesity is through the inhibition of enzymes such as pancreatic lipase [28,29,30], amylase, and glucosidase [31] in the gastrointestinal (GI) tract. Inhibition of lipase in the GI tract, with subsequent reduced fat absorption, is a well-known target for obesity treatment, with orlistat exploiting this very mechanism [32], whereas inhibition of amylase and glucosidase prevents digestion and absorption of carbohydrates, again reducing energy intake. 

The composition of gut microbiota is highly correlated with obesity and related diseases such as T2D [33,34,35], as intestinal bacteria have been shown to affect fat storage, blood glucose balance, and appetite hormones [36,37]. GT may influence the gut microbiota through two modes of action. Amylase and glucosidase inhibition increases the presence of undigested carbohydrates in the GI tract, in turn driving the microbiota to produce short-chain fatty acids (SCFA) [38,39], recently found to be capable of activating AMPK, and inducing weight-loss [40] through lipogenesis and lipolysis down- and up-regulation, respectively [41]. Moreover, most tea polyphenols (>90%) will pass through the small intestine unabsorbed due to their low bioavailability, eventually coming into direct contact with the gut microbes. These are capable of breaking them down into smaller and more bioavailable phenolic components, and in turn are modulated in terms of bacterial composition [42]. Although no direct evidence is available to date attributing GT-induced weight loss to gut microbiota modulation, it is reasonable to assume that this might be one of the possible underlying mechanisms.

The effectiveness of GT in reducing body weight and fat is widely discussed in the literature. GT derived EGCG in variable quantities (100–460 mg/day) exhibits measurable weight-loss properties in a large majority of studies according to a recent review and one meta-analysis, especially for trial durations of three or more months [43,44]. In addition, the consumption of caffeine at doses between 80 and 300 mg/day has been shown to be an important factor for these effects, when the participants did not have a high baseline caffeine intake (>300 mg/day). A recent eight-week study investigated the effects of green tea extract (GTE) supplementation on exercise-induced changes in sedentary, overweight women, showing that GTE improves exercise-induced body composition changes by decreasing weight, BMI, waist to hip ratio (WHR), and body fat percentage (BFP). Interestingly, there seems to be an ethnicity-dependent effect, with more important weight loss (mean 1.51 kg) in Asian subjects [27], compared with one of 0.82 kg in Caucasians [45]. However, not all evidence suggests a beneficial effect. According to a very recent meta-analysis, there is no evidence that GT or EGCG have a beneficial effect on maintaining weight loss [46]. 

In conclusion, GT, alone or in association with other weight loss interventions, seems a possibly useful tool for the treatment of obesity with close to no side effects, and evidence supporting its consumption is of moderate quality. The exact reason behind the presence of controversial results is yet to be elucidated: it has been hypothesized that one possible motivation might be the use of relatively low doses of EGCG (i.e., 200 mg/daily), but some studies investigating the effect of low amounts still reported positive outcomes [47]. Ethnicity, baseline caffeine intake, duration of obesity, dietary habits, the gut microbiota, and other inter-individual variabilities, as well as trial duration and co-interventions, might explain some of the observed inconsistencies in the data [26,43]. It would therefore be useful to deepen the research and evaluate, in particular, the appropriate dosage and the patient profile possibly benefiting the most from GT consumption.

#### 3.1.2. Ginseng

Ginseng refers to different varieties of a short, slow-growing plant with fleshy roots belonging to the *Araliaceae* family. The two main types of ginseng are *Panax ginseng* (Asian ginseng), also known as Korean Ginseng and *Panax quinquefolius* (American ginseng). Asian ginseng can be red or white depending on the drying method of the root [48]. Different ginseng extracts are being studied for the treatment of several medical conditions, including body weight management.

Ginseng is supposed to contribute to weight loss through its elevated content of saponins which can delay the intestinal absorption of dietary fat by inhibiting pancreatic lipase activity [49,50]. Moreover, ginseng intake may affect serum levels of leptin, adiponectin, and ghrelin, as demonstrated in obese mice after the administration of Korean ginseng whole extract (8–18 g/kg) for eight weeks [51].

Although *P. ginseng* has been shown to exert anti-obesity effects in several animal studies, there have been relatively few studies investigating its effects in humans. In a randomized, double-blind placebo-controlled trial, 24 women with obesity were administered 18 g of Korean red ginseng (KRG) for 8 weeks and showed a decrease in BMI, aspartate aminotransferase, food intake, waist-to-hip ratio, and improved quality of life, but a frank superiority over placebo was not confirmed [52]. Another trial conducted in patients with T2D confirmed that a much smaller supplementation of KRG (100–200 mg/day) had a positive impact on glucose levels, despite failing to induce significantly more weight loss compared to placebo [53]. A more recent line of evidence suggested that the administration of 6 g of KRG over 12 weeks had no significant effect on weight, BMI, fat mass, glucose, insulin, and levels of cholesterol when compared to placebo group [54]. Reeds et al. obtained similar results in a randomized control trial comparing the effect of ginseng and its active component *ginsenoside Re* in overweight/obese subjects with impaired glucose tolerance or newly diagnosed T2D. However, this study did not use body weight as a primary endpoint [55].

In conclusion, the clinical relevance of ginseng as a weight loss aid remains uncertain, as the evidence quality supporting its use is low and the dose range very high (100 mg–18 g/day). Further investigation comparing the effects of the two main types of Panax ginseng is also necessary.

#### 3.1.3. White Kidney Bean

The white kidney bean is one species of *Phaseolus vulgaris* L., also known as common bean, originating from South America. White Kidney Beanis rich in proteins (22–27% of seed weight) and carbohydrates (39–47% of seed weight), with a high content of bioactive compounds, such as peptides, among which are the α-amylase inhibitor named phaseolin, polyphenols, oligosaccharides, and lectins. Notably, the significant amount of lectins also arose some concerns, as, alongside their potential anti-cancer and anti-obesity activities, these peptides may also act as toxins and allergens [56,57]. Several reports are available regarding clinical adverse effects after ingestion of white kidney beans [58]. 

Conversely, *Phaseolus vulgaris* extracts (PVE) have been developed to isolate the action of phaseolin, an α-amylase inhibitor (α-AI) capable of binding to α-amylase non-covalently interfering in the breakdown of complex carbohydrates [59] with subsequent impaired absorption of these nutrients through the gut wall [60]. PVE are characterized by antioxidant, anticarcinogenic, anti-inflammatory, glucose lowering, and cardioprotective properties, together with potentially inducing weight loss [61,62,63]. Noteworthy, α-AI activity is highly dependent on pH, temperature, incubation time and the presence of specific ions, all of these having been optimized in some PVE commercial products, such as Phase2^®^ water extract of Phaseolus vulgaris standardized to alpha amylase (8;12;15;39) inhibiting units (Pharmachem Laboratories, Kearny, NJ, USA) [64]. Barrett et al. systematically reviewed ten studies conducted between 2000 and 2010 evaluating the effect of Phase 2^®^ products on body weight and glycemic control in subjects with overweight and obesity, demonstrating significant weight loss when the product was taken concurrently with carbohydrate containing meals. A recent metanalysis confirmed Phase2^®^ PVE efficacy on body weight and fat change [65], results being confirmed by a recent Chinese RCT involving 120 subjects with obesity, in which the group treated with PVE capsules experienced a mean placebo adjusted weight loss of 1.95 kg after 35 days [66]. Conversely, another metanalysis conducted by Onakpoya et al. including all kinds of PVE commercial products concluded for a substantial absence of efficacy of these towards weight loss outcomes [67].

The evidence supporting the use of PVE 1 to 3 g/day for weight loss, and especially that of Phase 2^®^ products, is of moderate quality, and its efficacy was demonstrated of sufficient clinical importance. PVE supplementation could be encouraged as a tool for weight loss.

#### 3.1.4. Chitosan

Chitosan is a natural polysaccharide of β-1,4-linked glucosamine residues and derives from deacetylation of chitin, the second most abundant biopolymer on the planet, mostly found in shrimp and crabs [68]. Thanks to its well-established beneficial effects on health and its favorable safety profile, the European Food Safety Authority (EFSA) Panel on Dietetic Products, Nutrition, and Allergies (NDA) recommended a maximum intake of 3 g of chitosan per day [69].

In particular, chitosan has been proposed as dietary supplement for the management of obesity because of its cholesterol-lowering properties. In fact, chitosan forms hydrophobic bonds with dietary cholesterol, therefore interfering with its emulsification and absorption [70]. Moreover, chitosan has been shown to decrease lipid peroxidation in rats fed a diet enriched with cholesterol, suggesting a possible antioxidant role [71]. Finally, in vitro studies have demonstrated that chitosan can modulates adipokine secretion and inhibit adipogenesis [72,73].

Chitosan has also been used in human studies. A recent metanalysis evaluated its effect in obese and overweight patients, including 14 studies conducted from 1999 to 2017. A total of 1101 participants were randomized, of whom 570 were allocated to chitosan and 531 allocated to placebo. The mean trial duration was 17 weeks (range 4–52 weeks), mean study size was 79 participants (range 12–250). Chitosan supplementation ranged from 1 to 4.5 g/day and a mean decrease in BMI of −1.27 kg/m^2^ in favor of chitosan versus placebo was observed [74].

Given the low-quality evidence and the poor clinical importance of its consumption, but considering the mild if any adverse events, chitosan consumption cannot be encouraged nor discouraged as obesity treatment at this time.

#### 3.1.5. β-Glucans

β-Glucans are natural bioactive fibers or polysaccharides composed of D-glucose monomers, linked by 1,3, 1,4, or 1,6 β-glycosidic bonds. They are naturally occuring in the cell wall of bacteria, fungi, algae, and higher crops, such as cereals and can be taken orally as a food supplement or as part of a daily diet [75]. The study of the effects on health of β-glucans is complicated by the variability of their biological activities which depend on the source, the extraction and purification methods.

Numerous pre-clinical and clinical studies have described the antitumor, antimutagenic, immune-modulating, anti-osteoporotic and antioxidant effects of β-Glucans. Moreover, they gained nutritionists attention because of their positive activities on glucose and lipid metabolism. In fact, when glucans are included in a meal, the rate of carbohydrate and lipid absorption slows down, ultimately leading to a decrease in plasma glucose and lipids [73,75,76,77,78,79,80]. Alongside the well-established anti-diabetic and lipid-lowering properties, preclinical studies also suggest that glucans may exert anti-obesity effects by activating the gut-hypothalamic (Peptide YY- Neuropetide Y) axis, therefore increasing satiety in diet-induced obese mice [81]. A dose-dependent increase in peptide YY levels has also been demonstrated in overweight adults following oat β-Glucans ingestion, suggesting that glucans may exert anorexigenic effects in humans as well [82]. At the moment, there are no clinical trials that have evaluated the effect of β-Glucans on weight loss other than one placebo controlled study in which overweight women followed a low-calorie diet plus β-glucans supplementation showing a similar decrease in body mass [83].

While evidence supporting the use of β-Glucans 5–9 g/day as anti-diabetic and lipid-lowering dietary supplement are promising, data investigating their effects on body weight are lacking and uncertain, so no recommendation can be made regarding their use as weight loss supplements.

#### 3.1.6. Psyllium

Psyllium is a water-soluble fiber derived from the husks of seeds from *Plantago ovata*, an officinal plant native to western and southern Asia. A soluble fiber has the ability to dissolve in water, forming a viscous gel that may decrease appetite by occupying the stomach and it may interfere with the absorption of carbohydrates [84,85], lipids and bile acids [86,87]. Some evidence suggests that psyllium may be capable of lowering serum lipids, delaying gastric emptying, improving glycemic control, and promoting satiety [88]. However, little is known relative to the role of psyllium in aiding body weight loss. 

Two literature revisions have investigated the effectiveness of psyllium in inducing weight loss, showing that its consumption may exert some beneficial effects in reducing body fat, especially in long-term clinical studies with a duration between 6 and 12 months, when its intake was associated to a dietary program and lifestyle modifications [88,89].

More recently, Xiao et al. conducted a metanalysis on four RCTs evaluating the effects of psyllium on body mass index, showing that BMI and body weight remained unaltered by psyllium intervention, although the results may be partially attributable to the low number of studies included [90]. Nevertheless, the discordance between the metanalysis conducted by Xiao et al. and the previous revisions may be explained by the different selection criteria adopted. In fact, Xiao et al. included only placebo controlled RCTs evaluating the effect of psyllium without other treatments, whereas the two previous revisions also included non-randomized trials, in which dietary psyllium was associated with other interventions.

Another recent metanalysis investigated the effects of psyllium consumption (whether prescribed through supplements or added to foods) on BMI and/or weight, including RCTs, both parallel and crossover, not necessarily placebo-controlled. From the metanalysis of the eleven studies (involving a total of 632 participants) reporting BMI as outcome, a non-significant reduction in BMI was observed, although subgroup analysis showed that psyllium supplementation at higher doses (≥10 g/d) significantly decreased BMI in studies with a duration ≥10 week [91].

Overall, the quality of evidence in literature evaluating the effects of psyllium consumption on weight loss is low and limited by the great heterogeneity between different studies in terms of duration, design, type of intervention, and dose utilized. Therefore, the use of psyllium supplementation for inducing weight loss cannot be recommended at the moment.

#### 3.1.7. Glucomannan

Glucomannan is a polysaccharide composed of β-1,4–linked D-mannose and D-glucose monomers which comes from the tuber *Amorphophallus konjac*. It is a soluble fiber which is present naturally and abundantly in several products, such as softwoods, roots, tubers, and many plant bulbs [92]. It has been suggested that the ingestion of glucomannan could determine the prolongation of gastric emptying time, thus resulting in increased satiety and decreased post-prandial glucose concentration [93,94]. Moreover, glucomannan is able to absorb up to 50 times its weight in water and cannot be digested by human salivary and pancreatic amylase so that it passes relatively unchanged into the colon, where it is fermented by the gut microbiota [95].

Two meta-analyses supported the role played by glucomannan in weight loss. According to Keithley et al., glucomannan administration at doses of 2–4 g per day resulted in significant weight loss in overweight and obese individuals [95]. Moreover, in a metanalysis of 14 RCTs, Sood et al. suggested that patients receiving 1 g glucomannan daily, together with 1 glass of water and an energy-restricted diet, showed statistically significantly lower total cholesterol, LDL cholesterol, triglycerides and body weight when compared to placebo. Of note, paediatric patients, or subjects with impaired glucose metabolism did not benefit from glucomannan administration to the same degree [96,97]. Noteworthy, a prospective, non-randomized controlled trial suggested a possible beneficial role of glucomannan on weight loss in overweight subjects treated with a combination of Garcinia Cambogia and glucomannan at a dosage of 500 mg, twice a day, for six months. However, the administration in association with Garcinia Cambogia made the individual effect of GMM indistinguishable [98]. Finally, Onakpoya et al., in a systematic review and metanalysis of nine trials, indicated that glucomannan intake does not generate a significant decrease in body weight or BMI when compared to placebo [99].

In conclusion, the clinical relevance of glucomannan as a weight loss aid remains uncertain and the quality low. Further investigation through larger studies is necessary. At the moment, the consumption of glucomannan for the treatment of obesity should not be encouraged nor recommended against.

#### 3.1.8. Guar Gum

Guar gum, also known as “guaran”, is a fiber derived from the seed of *Cyamopsis tetragonolobus*, an Indian leguminous plant. From a molecular point of view, guar gum is a complex polysaccharide called galactomannan, which is a polymer of D-galactose and D-mannose. It is widely used as an additive in food, in the form of guar gum powder. Guar gum can be also found in several types of food such as dairy products, cereals, sauces, pudding, kefir, and baked goods [100].

It has been suggested that guar gum may contribute to lower body weight by increasing the viscosity of the bowel content and the feeling of postprandial fullness, thus reducing appetite and food intake [101]. 

Pittler et al., in a systematic review and metanalysis of 11 trials, indicated no statistically-significant difference in patients receiving a supplementation of 9–30g/day of guar gum for up to 6 months, compared with those receiving placebo upon continuation of the usual dietary habits in most studies [102]. Noteworthy, in a double-blind, placebo-controlled trial included in the previous metanalysis, patients with T2D receiving up to 21 g/day of guar gum reported a placebo-adjusted body weight loss of 3 kg and a total cholesterol decrease of 11% [103]. These findings suggest a reduction in the risk for cardiovascular complications in diabetic patients after guar gum supplementation. Another more recent line of evidence showed that the intake of guar fiber alone at a dose of >5 g/serving or its combination with protein (2.6 g guar fiber + 8 g protein/serving) led to acute satiety effects in normal weight subjects, but did not affect body mass [104]. The most frequently reported adverse effect of guar gum supplementation was GI discomfort, such as flatulence, diarrhoea, and abdominal pain, occasionally leading to drop-out from studies [102].

These results suggest that, although guar gum can lower cholesterol levels and improve insulin sensitivity compared with placebo [105], it seems not to be effective in lowering body weight and it should therefore be administered together with appropriate dietary manipulation. 

#### 3.1.9. Agar

Agar is a gelatinous substance extracted from a type of red algae, agarophytes; it is a fiber-rich mixture of natural polysaccharides, mainly agarose and agaropectin. Agar has the ability to melt to sol at temperatures greater than 85 degrees and form a gel when cooling a hot solution to 30–40 degrees, therefore it falls in the water-soluble fiber category [106]. Agar has been used in Japan and other Asian countries for many years to make desserts such as jellies, puddings, custards, and bubble tea. It is also used as a thickener for soups and ice creams and as a gelling agent for fish and meat-based products. Moreover, given its resistance to be enzymatically degraded by most bacterial species, agar is also used in microbiology as a substrate for culture media and, because it is not degraded by the human GI tract, it has also been used as a laxative.

Agar has been shown to prolong gastric emptying [107,108], thus increasing fullness sensation [107]. Surprisingly, the addition of agar to a test meal was shown to increase other appetite parameters such as hunger and desire to eat [108], questioning its ability to inhibit appetite and caloric intake; no positive effect was also seen on energy expenditure after the test meal.

A 16-week long study by Maeda et al. on 76 patients with T2D or impaired glucose tolerance, showed that the addition of 180 gr of agar to a conventional Japanese diet led to a greater reduction of body weight (4.4% vs. 2.0% in non-agar diet) and BMI (1.5% vs. 1.0%), along with a reduction of total body fat, measured by dual-energy X-ray absorptiometry /DXA) scan, and of visceral and subcutaneous fat areas measured by computed tomography (CT) at the umbilical level; the greater weight reduction in agar diet group was supported by a greater reduction in the total daily calorie intake, especially at the evening meal, suggesting a possible appetite suppressant property despite appetite related parameters not being analyzed in the study. The agar diet also led to an improvement in glycometabolic parameters with a significant reduction in HbA1c, fasting glucose and total cholesterol [109], and no safety concern emerged.

Overall, the quality of evidence supporting the use of agar for weight loss is low, given the absence of well-designed studies in normoglycaemic obese patients and the paucity of data regarding weight loss in patients with alterations in glucose metabolism. Although current evidence is consistent with other studies that analyzed weight loss with soluble fiber rich diets, suggesting a possible positive effect of agar on weight reduction, there is not enough evidence to recommend its use for this purpose [110].

#### 3.1.10. Inulin and Inulin Type Fructans

Inulin is a polysaccharide produced by many plants and extracted principally from chicory. It is part of the inulin-type fructans (ITFs) family which covers all β (2←1) linear fructose polymers, such as native inulin, oligofructose and fructo-olysaccharides (FOS). These compounds are resistant to digestion and undergo a selective fermentation, thus acting as dietary fiber and bifidogenic prebiotic [111,112]. Inulin is also used as a fat and sugar replacement and texture modifier in many bakery, dairy and meat products [113].

ITFs have been shown to be capable of regulating GI hormones release in both animals [114] and humans [115]: inulin and FOS supplementation are able to increase Gucagon like petide -1 (GLP-1) and PYY release and suppress ghrelin secretion; furthermore, the prebiotic properties of ITFs may modulate the gut microbiota, favoring the growth of beneficial bacteria such as short-chain fatty-acids (SCFA) producers, thus improving satiety and weight loss and reducing systemic inflammation [116]. 

Whether these proposed mechanisms of action are able in turn to suppress appetite and promote weight loss is a matter of debate: the authors of a 2013 review analyzed the effect of different doses of ITFs, ranging from 8 to 21 gr/day, on appetite, energy intake and weight loss in an adult non-diabetic population. Of the 15 RCTs included in the literature revision, none described positive effects regarding appetite suppression and daily energy intake reduction except for one study, and only two showed a significant weight reduction in subjects with obesity, with one of these observing a concomitant reduction in total energy intake [117]. Pointing in the same direction, two recent RCTs and one crossover double blind study did not report any additional weight loss upon consumption of 9–10 g of inulin alone or with 10 g of maltodextrin in a non-diabetic population with overweight or obesity following a hypocaloric diet, although positive effects on blood pressure and cholesterol were recorded [118,119], suggesting that inulin fails to promote additional weight loss when subjects are following a controlled hypocaloric diet. Interestingly, fat mass loss was also observed in one of these studies [119]. A recent review analyzing 12 RCTs from 2013 to 2015 described positive effects of 10 gr inulin or FOS-enriched inulin on weight reduction, satiety, and daily energy intake, mostly in patients with T2D, in addition to greater reductions in Hba1c and other metabolic syndrome related parameters [116]. However, studies on non-diabetic patients with obesity showed, consistent with previous evidence, discordant results, despite positive gut microbiota modulations being observed. These discrepancies could be partly explained by the more marked metabolic alterations in T2D and the potential greater effect of prebiotics on gut microflora in these subjects. Another longer trial conducted on prediabetic subjects and testing higher doses of inulin (30 g) co-administered with a hypocaloric diet, followed by ad libitum food consumption, showed that similar weight loss was observed compared to placebo during the hypocaloric regimen phase, but weight loss became significantly more pronounced during the ad libitum phase in those taking inulin [120]. These findings suggest that inulin may be able to reduce daily caloric intake possibly promoting fullness and reducing hunger and may therefore be beneficial when food intake is not restricted.

Overall, most evidence shows that inulin and ITFs supplementation has a positive effect in subjects with T2D regarding weight loss, appetite suppression, glucose metabolism and systemic inflammation parameters, while such effects have not been described consistently in those with obesity but no diagnosis of T2D. Noteworthy, the quality of evidence supporting the application of inulin for weight loss is low, considering that most of the analyzed studies are heterogeneous in terms of interventions (inulin and FOS dosages and method of administration, hypocaloric or ad libitum diets), study population (normal weight, patients with obesity or T2D), trial duration, and outcomes analyzed, which may hinder the strength of the results. Better quality studies are therefore needed to fully elucidate the effect of these compounds and to evaluate whether the results described are different based on the type of compound used and if they really are magnified in patients with altered glucose metabolism. Moreover, daily caloric intake should be closely monitored to determine if the weight loss obtained is the result of a reduced daily caloric intake or the effect of changes in gut microbiota and GI hormones milieu. 

At this time, inulin should be not recommended for the treatment of obesity, although it may be considered in obese patients suffering from T2D.

### 3.2. Reduced Appetite as Purported Mechanisms of Action

One of the major obstacles to weight maintenance is the decreased satiety that derives from weight loss and hypocaloric diets. Hence, safe food supplements that may decrease appetite can find a role in the treatment of weight excess. Herein, we provide detail on those that have the strongest evidence in inducing satiety in human studies, those that act on appetite through mechanic distention of the bowels have been included in the previous section as they also inhibit absorption of nutrients in a steric fashion (Table 2).

#### 3.2.1. Caralluma

Caralluma Fimbriata is an edible succulent plant belonging to the *Asclepiadaceae* family. It is found in Africa, India, Arabia, and Southern Europe [121]. Pregnane glycosides, the main bioactive compounds, are thought to be the ones responsible for the reported appetite-suppressant and weight loss inducing properties of caralluma. This seems to be obtained through citrate lyase and malonyl coenzyme A inhibition that in turn leads to inhibition of fatty acid synthesis and enhanced fatty acid oxidation [122]. Furthermore, caralluma glycosides also seem to reduce appetite by acting on the hypothalamus through the amplification of energy sensing function signalling or through the inhibition of ghrelin/neuropeptide Y expression [121]. A preclinical study investigating the anti-obesogenic effect of caralluma showed that rats supplemented with it reported a significant reduction in food intake, an inhibition of body weight, liver weight and fat mass gain [123].

Two RCTs conducted on obese and overweight subjects showed a significant reduction in weight and waist circumference after treatment with caralluma 500 mg twice daily for two months [124,125], whereas one other did not find significant changes in body weight and BMI after treatment with the same dosages and longer time (three months) [126]. No major side effects were reported at the dosages utilized in the human studies.

Given the scarcity of data and the low-quality of the evidence on the anti-obesogenic effect of caralluma in human subjects, it is not possible at this time to recommend its use despite it being reasonably devoid of side effects. 

#### 3.2.2. Spirulina

Spirulina is a cyanobacterium, belonging to the *Athrospira* genus. Some species of spirulina are used as food supplements, as they represent rich sources of high quality and almost all essential amino acids, essential polyunsaturated fatty acids like α-linolenic acid, eicosapentaenoic acid (EPA), docosahexaenoic acid (DHA), minerals, vitamins, and antioxidants including phycocyanins, carotenoids, tocopherols, and phenolic compounds [127]. The role of spirulina on body weight control is not fully elucidated yet. In murine models, spirulina extracts administration showed anti-obesity and lipids-lowering effects [128,129], mediated by different mechanisms, such as adipogenesis suppression, browning of white adipose tissue [129], and modification in brain and liver genes expression [128]. In humans, it proved effective in decreasing appetite [130]. 

Clinical studies evaluating the effects of spirulina consumption on weight reduction are limited. A recent metanalysis investigated the effects of spirulina supplementation on body weight reduction, including only five RCTs, published between 1996 and 2018, and 278 patients overall, consuming doses of spirulina between one and 4.5 g/day, for a duration from six to 12 weeks. An overall significant reduction in body weight (mean −1.56 Kg) body fat percentage and waist circumference were observed, with no change in BMI and waist-to-hip ratio [131]. However, the analysis was limited by the small number of studies included. A successive placebo controlled RCT evaluating the effects of consumption of 4.5 mg/day of spirulina extract in addition to a physical exercise program for six weeks on 45 overweight and obese men did not find any significant changes in anthropometric parameters, whereas it confirmed the lipid lowering effects [132]. 

Overall, despite its promising effects described in animal models, evidence supporting the use of Spirulina for weight loss purposes in humans is scarce, controversial and of low quality. Its supplementation should therefore not be recommended as a treatment for obesity at this time.

#### 3.2.3. Whey Protein

Whey protein (WP) is the water-soluble part of milk and it is considered to be a high biological value protein, including all essential 22 amino acids. WP is usually available in powder form and can be easily added to beverages and some foods. Consumption of WP has been shown to increase circulating concentrations of satiety hormones of the lower gut, including GLP-1 and PYY thus suppressing appetite more than other proteins, such as casein, soy, and egg albumin. Moreover, WP may promote fat mass reduction through its oxidation, with concomitant preservation of lean mass [133].

A systematic review and metanalysis of 14 RCTs examined the effect of WP, with or without resistance exercise, on body weight and body composition. Five studies analysed the effects of WP as a replacement for other sources of calories (WPR) and the remaining nine studies examined the effects of WP as a supplement without dietary modification, and body weight and fat were significantly decreased from baseline in the WPR within-group analyses [134]. Similarly, another more recent metanalysis assessed the impact of WP supplementation in its concentrated (WPC), hydrolysed (WPH) and isolated (WPI) forms, comparing it to isocaloric placebo in athletes. WPC was the only form with a statistically significant impact on fat mass loss [135].

In conclusion, evidence supporting the use of WP 100–600 g/week, either as a supplement combined with resistance exercise or as part of a dietary restriction program in order to improve body composition parameters, is of moderate quality, but further dose/response trials should be performed. Overall, WP supplementation may be recommended for the treatment of obesity.

#### 3.2.4. Coffee, Caffeine and Chlorogenic Acids

Coffee is one of the most popular beverages consumed worldwide, prepared from roasted coffee beans. Scientific studies often lack an adequate differentiation between the effects of its single compounds, whose most important are caffeine, chlorogenic acid (CGA), diterpenes, and trigonelline. Caffeine is a naturally occurring alkaloid with well-established stimulating properties. As a lipid soluble compound, it freely crosses the blood brain barrier [136], and, therefore, affects neural function. Caffeine appears to suppress hunger and stimulate energy expenditure (EE) through increased excitability of the sympathetic nervous system (SNS), increased fat oxidation and Brown Adipose Tissue activation [137,138]. CGA is an antioxidant and anti-inflammatory phenolic acid playing a role in neuro- and hepatoprotection, and with lipid and glucose lowering properties [139,140]. 

A recent systematic review and dose-response metanalysis highlighted the positive effects of caffeine consumption on weight and fat loss, taking into account studies published between 1999 and 2014 [141]. However, among the thirteen studies selected, most had caffeine being administered in association with other compounds, such as ephedrine or green tea, or in the form of coffee beverage, making the effects of caffeine from CGA and other elements indistinguishable. One study conducted on 30 subjects with obesity and lasting 12 weeks showed greater weight loss upon consumption of instant coffee rich in CGA and caffeine compared to decaffeinated with similar amounts of CGA [142]. Similarly, Davoodi et al. demonstrated greater weight loss in 15 women with obesity taking an oral solution of 5 mg of caffeine/Kg/day for four weeks compared to controls following the same diet without caffeine consumption. The caffeine-treated group demonstrated suppressed hunger and continued to lose weight during the one-month-follow-up period, whereas the controls slightly increased body weight [143].

Several studies have evaluated how green coffee extracts, rich in both CGA and caffeine, have proven effective in inducing weight loss [140]. Interestingly, in a cross-over study, 20 volunteers were subjected to the consumption of green coffee (richer in CGA) and black coffee, with a wash-out period in between, showing that BMI fell significantly only upon green coffee intake, whereas both beverages significantly reduced abdominal fat despite constant energy intake and physical activity throughout the intervention.

Overall, moderate-quality evidence suggests that black and green coffee, and both caffeine and CGA, may potentially induce weight loss, and they should be considered as a tool for the treatment of obesity. However, further studies are needed to better evaluate the effects of the single components on body weight.

#### 3.2.5. Bitter Orange

Bitter orange, the fruit of the *Citrus aurantium*, is rich in p-synephrine, a primary protoalkaloid which has been widely used for weight-loss management as suppressor of appetite and stimulator of energy expenditure and lipolysis [144,145]. P-synephrine has some structural similarity to ephedrine, from which it differs by the presence of a hydroxyl group in the para position on the benzene ring [146]. 

Bitter orange extracts’ efficacy in inducing weight loss has been shown in some clinical studies [147,148], and literature revisions including studies conducted on subjects who received products containing p-synephrine alone or in combination with other supplements concluded that the consumption of this dietary supplement is overall safe and may induce modest weight loss [149,150]. The most recent review, involving approximately 30 human studies and over 600 subjects, have confirmed that evidences supporting the anti-obesogenic role of bitter orange extracts are limited and uncertain, as they are often studied in combination with other molecules. P-synephrine does not appear to produce cardiovascular effects at doses up to 100 mg [144]. Human clinical trials evaluating the effects of bitter orange extracts on weight outcomes have not been published since 2016, although some recent preclinical study showed that Citrus peel extracts attenuated obesity and modulated gut microbiota in a high-fat diet-induced obesity mice [151] and regulated in vitro adipogenesis and thermogenesis via AMPK activation [152].

At the moment, bitter orange should not be recommended for the treatment of obesity as the quality of evidence supporting its application for this purpose is low.

#### 3.2.6. Guarana

Guarana is a native plant from the Amazon, where it is traditionally brewed as a drink. Its fruit and seed are rich in catechins and methylxantines, that inhibit adipogenesis [153], promote browning in animal models [154], and stimulate energy expenditure in humans [155]. Their use as nutraceutical supplement for weight loss and as stimulant is widely spread. 

Little evidence in human subjects suggests a possible efficacy in inducing weight loss [156] and in protecting from metabolic syndrome [157] when used alone, with dosages of 240–285 mg/day. Conversely, its combination with other supplements is reported to delay gastric emptying leading to increased satiety and significant weight loss [158]. Possible side effects have been reported, generally observed when herbal supplements associations were being used [159]. 

Despite the promising preclinical evidence, the paucity of data and the low-quality evidence regarding the effects of guarana consumption on weight loss in human subjects makes its potential application as therapeutic agent to treat obesity uncertain, so its consumption should be not encouraged to induce weight loss.

### 3.3. Increased Energy Expenditure as Purported Mechanisms of Action

The fascinating hypothesis that natural and harmless substances could increase energy expenditure has been going on for the past several years. In the 1960s, the family of amphetamines was very popular among those willing to lose weight but was later abandoned due to several associated cardiovascular accidents. Thyroid hormones followed the same pathway, and since then, the scientific community has tried to find the “perfect” stimulant. Fibroblast growth factor 21 was recently tested on human beings, as it was shown to induce BAT activation and browning of white adipose tissue in rodents, with a net effect of increased energy expenditure [160,161]. However, studies on primates and human subjects quickly proved that the physiology of energy metabolism is very different across species, and the same effect could not be replicated in men or women. Here, we provide more details concerning natural compounds proven to exert an effect on energy expenditure, although it should be kept in mind that most mechanistic evidence is on rodent models, and the weight loss effect only was proven in human subjects. It is therefore not possible to conclude that the same mechanism of action observed in preclinical studies is the one responsible for the anti-obesogenic effect in human beings (Table 3).

#### 3.3.1. Capsaicin, Capsaicinoids and Capsinoids

Capsaicin is an active compound of chili peppers and a component of the capsaicinoids family [162]. Besides its use in food preparation, it is used as an analgesic in ointments and patches for neuralgia and neuropathy treatment [163]. Capsinoids, also present in chili peppers, are structurally similar to capsaicinoids and bind to the same receptor, but they do not have the same characteristic pungency because they cannot reach mouth receptors due to their structural difference [164].

Capsaicin effects are ascribable to the activation of transient receptor potential channel vanilloid type-1 (TRPV1) [165], causing an influx of Ca^2+^, and subsequent release of neurotransmitters, such as substance P and catecholamines, and other substances like GLP-1 [166]. In animal models, capsaicin has also been shown to suppress ghrelin release [166], increase adiponectin mRNA expression in the adipose tissue and PPARα/PGC-1α mRNA in the liver [167], enhance AMPK and regulate gluconeogenesis and glycogen synthesis genes [168], thus reducing obesity-induced insulin resistance. Other studies have demonstrated that capsaicin and capsaicinoids are able to induce thermogenesis, activating UCP-1 and 2 [169], increase fat oxidation, SNS activity, and energy expenditure via GI TRPV1 activation [170] and promote SIRT-1 expression, inducing browning of white adipose tissue [171].

A 2012 review of twenty clinical trials investigated the effects of capsaicinoids and capsinoids on energy expenditure, lipid oxidation and appetite regulation, in both short term (meal tests) and long-term interventions (up to four months). Capsaicinoids doses ranged between 10 and 36 mg/day, while trials assessing the effect of capsinoids used less than 10 mg/day. Most trials showed a beneficial effect of both capsaicinoids and capsinoids on energy expenditure, with a small increase in oxygen consumption, body temperature, and metabolic rate (about 50 kcal/day). Furthermore, capsaicinoids and capsinoids were able to increase satiety and reduce hunger, thus decreasing energy intake, in five of the included studies [172]. In a 2014, a systematic review and metanalysis analyzing capsaicin and capsaicinoids effect on weight management, capsaicinoids consumption before meals resulted in a significant reduction in ad libitum energy intake (74 kcaL/meal) with significantly higher reduction for dosages over 2 mg [173]. Subgroup analysis, however, showed high heterogeneity, even when only trials using high capsaicinoids doses were taken into account, suggesting high variability in trial designs. A recent metanalysis showed that ingestion of capsaicin or capsinoids leads to increased energy expenditure (58.56 kcal/day) and decreased respiratory quotient, indicating enhanced fat oxidation. Interestingly, subgroup analysis demonstrated an increase in energy expenditure (69.79 kcal/day) and a decreased respiratory quotient only in subjects with BMI > 25 and a significant effect only in study with short duration (<1 day) [174]. These results indicate that capsaicinoids and capsinoids consumption may induce a tolerance mechanism long term and may have a BMI dependent enhancement of fat oxidation and energy expenditure. As the authors reported, the latter may be partly a consequence of the depressed SNS activity seen in obese subjects, promoting a positive energy balance [175].

The body of evidence shows that Capsaicinoids and capsaicin are effective in promoting a negative energy balance short term via thermogenesis enhancement and energy intake reduction, especially in overweight and obese subjects. However, whether this translates in sustained weight loss is still unclear and the quality of evidence supporting its application for obesity treatment is low. Studies conducted in the past showed that the ability to influence energy balance is not always successful in obesity management. Furthermore, long term studies analyzing capsaicinoids effect on energy expenditure failed to demonstrate a beneficial effect consistently, and higher quality studies are therefore needed to elucidate their potential on weight management. 

Despite the quality of evidence being classified as low, the strength of preclinical evidence and the promising results from human studies, together with the good safety profile, may support the use of capsaicinoids and capsaicin for the treatment of obesity.

#### 3.3.2. Curcumin

Curcumin is a bioactive polyphenol component derived from turmeric, a rhizomatous herbaceous perennial plant (*Curcuma longa*). It is a popular yellow spice often found in curry powder, claimed to play an important role against several pathological conditions such as atherosclerosis, cancer, and neurodegenerative diseases [176]. It has also been suggested that curcumin induces the secretion of adiponectin and inhibits adipocyte differentiation, together with possessing insulin-sensitizing and anti-inflammatory properties [177].

A double-blind, randomized, placebo-controlled trial reported that curcumin administration is well-tolerated and can positively influence weight management in overweight people by increasing weight loss (from 1.88% to 4.91%), and reducing body fat percentage (from 0.70% to 8.43%) [178]. In addition, a significant reduction of BMI and liver fat was also observed by Rahmani et al. after the administration of 70 mg/day curcumin for eight weeks among people with NAFLD [179]. Another more recent line of evidence is represented by a systematic review and metanalysis of 21 RCTs which indicated that curcumin intake significantly reduced BMI, weight, and waist-circumference [180]. However, not all reports point in the same promising direction. For instance, Mohammadi et al. did not find a statistically significant difference in anthropometric parameters such as BMI, weight and total body fat of obese patients receiving a supplementation of curcumin at a dosage of 1 g/day compared to placebo [181]. Similar findings have been reported by Ghazimoradi et al. after curcumin administration at the same dosage in patients with metabolic syndrome over six weeks [182].

Even though curcumin administration seems to be associated with significant reduction in body weight and body composition improvement, the majority of the RCTs only included a modest number of participants and were performed with unformulated curcumin which has very low bioavailability because of its poor absorption and rapid metabolism [183]. Moreover, curcumin supplementation is considered safe and no adverse side effects have been reported at low doses, while mild side effects such as digestive issues, headaches, nausea, or skin rashes have been rarely reported at higher doses (0.5–12g) [177,184].

Overall, evidence supporting the use of curcumin for weight loss are low in quality. Therefore, treatment with curcumin for weight loss purposes should be proposed only when a favorable cost-to-benefit ratio has been found and side effects should be looked for, especially when higher dosages are being proposed.

#### 3.3.3. L-Carnitine

L-Carnitine (L-C) is a naturally occurring amino-acid derivative, found in the majority of mammalian tissues including the brain. The liver and the kidneys produce sufficient amounts from the amino-acids lysine and methionine. However, some individuals (such as preterm infants), do not produce sufficient amounts, making L-C a conditionally essential nutrient. The best sources of L-C are animal products like red meat, fish, poultry, and dairy products [185].

L-C is reported to contribute to body weight reduction through a variety of different mechanisms. First, it is suggested to improve insulin resistance [186] and to stimulate energy metabolism in animal models [187] and fat oxidation in humans [188]. Moreover, it is also essential in facilitating activated long chain fatty acids transportation into mitochondria, playing an important role in β-oxidation; it seems capable of modulating regulators of lipid catabolism or adipogenesis such as hormone-sensitive lipase, acyl-coenzyme A oxidase, and carnitine palmitoyl transferase I-A [189]. Finally, it has been suggested that L-C supplementation may induce satiety [190].

Even though several preclinical studies demonstrate the efficacy of L-carnitine supplementation for weight management, findings in clinical trials are contradictory. On the one hand, two different trials reported no weight loss effect of L-carnitine supplementation in comparison with moderate aerobic training in obese women [191,192]. On the other hand, two different metanalyses showed that L-C supplementation can significantly decrease body weight and fat mass, although a decreased effect over time was reported [193,194]. In addition, a dose–response analysis showed that L-C supplementation changed BMI according to a non-linear function, with higher doses increasing the reduction [194]. Noteworthy, a specific subgroup analysis revealed that L-C exerts anti-obesity effects in overweight and obese subjects only [194]. Overall, L-C dosages in clinical studies are highly variable (10 mg–4 g/die), possibly influencing efficacy outcomes.

In conclusion, evidence supporting the use of L-C as drug for weight loss in adults is still of low quality, but given the strength of preclinical evidence and the promising results from human studies, together with the good safety profile, this supplement could be considered as a treatment of obesity. However, dose escalating trials must be performed to improve the evidence in this field thus finding the best and safest daily dose. 

### 3.4. Improved Fat Metabolism as Purported Mechanisms of Action

Obesity is defined by the World Health Organization as a condition of fat excess, rather than as a simple increase in BMI. It goes without explanation that no weight loss intervention is clinically relevant if it does not coincide with a reduction in body fat. The use of supplements claimed to “burn” fat is widespread, but only some of these have been proven to effectively and safely play a beneficial role on fat metabolism in preclinical studies. Of these, even fewer have shown such efficacy in human studies. Herein, we provide detail on those supplements supported by the most clinical evidence (Table 4).

#### 3.4.1. Pyruvate

Pyruvate (a derivative of pyruvic acid) is a physiologic breakdown product of body metabolism. It is a 3-carbon intermediate product of the glycolysis pathway and it can be converted to lactate or to acetyl-CoA in the cytoplasm or mitochondria, respectively. Natural sources of pyruvate are cheese, apples, and red wine. Its mechanism of action is unclear and only partially studied. It has been proposed that pyruvate may induce the shift in substrate utilization from predominantly carbohydrate to predominantly fat following pyruvate consumption, which may in turn contribute to increase fat oxidation, a mechanism to which both lower insulin levels and higher acetylCoA concentrations may contribute to [195]. The results of Ivy et al. indicated that chronic dietary supplementation with pyruvate reduced weight gain in obese Zucker rats in part by increasing resting metabolic rate and fatty acid oxidation [196]. Moreover, chronic consumption of pyruvate and dihydroxyacetone led to increased plasma thyroxin and decreased plasma insulin in rodent models [197].

A recent systematic review of clinical studies investigating the effect of pyruvate on body weight revealed a significant difference in body weight and fat loss favoring pyruvate (5–44 g/day) over placebo. However, the magnitude and the clinical relevance of this effect is small and uncertain, given that only six RCTs were included in the analysis and most had methodological weaknesses, with no study after 2005 being found by the reviewers [198]. The reason why there has been an abrupt interruption of studies assessing the effect of pyruvate on weight loss is unclear, as no safety concern emerged, and results were modest but promising. 

Given the lack of recent data and the low-quality of evidence in literature, pyruvate consumption should not be encouraged as a tool for inducing weight loss.

#### 3.4.2. Diacylglycerol

Diacylglycerol (DAG) is a natural component of several edible oils such as rapeseed and cottonseed oil [199], and is therefore easily added to foods. It is “generally recognized as safe” by the US FDA and has been allowed as a “food for specific health use” in Japan [200]. As several studies investigated the beneficial effects of DAG compared to Triacylglycerol (TAG), it is important to underline that DAG and TAG, despite similar energy density (38.9 kJ/g and 39.6 kJ/g, respectively) [201] and fatty acid composition, play different roles on lipid metabolism thanks to specific structural differences, namely the position of the fatty acid on the glycerol skeleton of DAG [202]. Indeed, DAG enhances fat oxidation and decreases the re-synthesis of chylomicrons in animal models [203], and its peculiar structural and metabolic characteristics seem to be responsible for the suppression of body fat accumulation, body weight loss and lower postprandial serum TG levels found upon the consumption of DAG-rich oils [204]. 

As highlighted by Rudkowska et al., data from several clinical trials suggest that 1,3-DAG is capable of decreasing body weight, visceral fat, and serum postprandial TAG concentrations, although results are controversial [205]. Moreover, Li et al. investigated the beneficial effects of DAG consumption on T2D patients, demonstrating an improvement not only in relation to body weight and waist circumference, but also regarding glucose metabolism and blood pressure compared with TAG [206]. Pointing in the same direction, the consumption of DAG together with alpha linoleic acid (ALA), a fatty acid undergoing easier beta oxidation as opposed to palmitic, stearic, oleic, or linoleic acid [207,208,209], resulted in excellent outcomes on body weight and visceral fat loss [203,210,211].

Overall, moderate-quality evidence suggests that DAG at a dosage of 1.1–1.2 g/day is effective in reducing body weight in both healthy and diabetic subjects compared to TAG, leading to a reduction in cardiovascular risk, with no adverse events being reported, and its use as a weight loss supplement could therefore be recommended. 

#### 3.4.3. Licorice

Licorice is a plant of ancient origin, deriving from the root of *Glycyrrhiza glabra* L. (Leguminosae). Its major compounds are: Glycyrrhizin (glycyrrhizic acid), used as flavoring agent; Carbenoxolone, a glycyrrhetinic acid derivative with a steroid-like structure which has been used in the treatment of peptic ulcer disease [212]; and different flavonoids, such as glabridin [213]. Licorice has been recently shown to exert beneficial effects on health; for example, it seems to be hepatoprotective in case of NAFLD [214] and contributes to improve the lipid profile in overweight and obese patents [215]. However, licorice consumption is also associated with the elevation of blood pressure, mediated via the mineralocorticoid receptor [216]. The way licorice extracts may contribute to body fat loss is still unknown, even if pre-clinical observations suggest that the anti-obesity effects could be mediated by glabridin, which has been capable of improving hepatic steatosis through beta-oxidation induction [217] and of ameliorating obesity via AMPK activation in high-fat-fed obese mice [218].

A recent metanalysis evaluated the metabolic changes associated with the consumption of licorice or its derivatives (300–900 mg/day), taking into consideration 26 clinical studies published from 2002 to 2017, with a duration ranging from two to 16 weeks. In particular, they verified that licorice consumption slightly but significantly reduced body weight dependent on the dose and duration of the treatment; the results also confirmed an increase of blood pressure and hypernatremia [219]. Notably, a recent eight-week long placebo-controlled RCT involving 64 overweight and obese patients did not show significant weight loss in the licorice-extract-treated group [220].

Overall, evidence supporting the use of licorice or licorice extract for weight loss is conflicting and of low-quality. Given the negative effects of licorice on blood pressure, and being hypertension a common complication in patients with weight excess, the use of this dietary supplement should be discouraged at this time.

#### 3.4.4. Garcinia Cambogia

*G. cambogia (GC)* is a tree native to the evergreen forests of India, Nepal, and Sri Lanka, where it is most often used for food and medicinal purposes. *GC* fruit extracts, known to cause watery diarrhea, have been used for constipation. Although numerous chemicals have been isolated from *GC* fruit, hydroxycitric acid (HCA) is considered the active ingredient for the anti-obesity properties that this dietary supplement has shown.

HCA, extracted from the rind of the fruit, is an organic acid which has been proved to be a potent competitive inhibitor of adenosine triphosphate-citrate lyase [221], therefore reducing the availability of acetyl-coenzyme A for fatty acids and cholesterol synthesis [222]. Pre-clinical studies also demonstrated that GC extracts attenuated fat accumulation through regulation of lipolysis genes expression via the adiponectin-AMPK signaling pathway [223]. Haber et al. reviewed nine randomized, double-blind, placebo-controlled trials, conducted from 1998 to 2014, involving the consumption of GC by individuals with obesity [224]. The results were controversial, considering that some studies did not show a significant difference in body weight [225,226,227] whereas others demonstrated that the supplement was more effective in decreasing body fat compared to placebo [228,229,230,231]. The controversial results may be explained by the presence of methodological weaknesses, such as short duration (2–12 weeks), variable dosages (400–2400 mg/day), and often lack of information such as sample size calculation. Although in short term trials GC has been proven as generally safe, it is noteworthy to underline that FDA urged consumers to avoid Hydroxycut, a natural product for weight loss containing GC and a variety of other ingredients, because of the report of twenty-three cases of hepatotoxicity associated with its use in 2009 [232]. Moreover, several hepatoxicity cases have been associated also with the use of pure GC extracts [233,234,235,236,237,238].

Overall, the use of GC supplements as treatment for weight excess is to discourage considering the uncertainty of its clinical efficacy and the safety concerns.

#### 3.4.5. Resveratrol

Resveratrol is a natural polyphenol found in a large variety of plant species such as grape, berries, and nuts, and has long been used as food supplement [239]. It has been reported to exert antioxidant, anti-inflammatory, and anti-carcinogenic effects, and it seems to be cardio- and neuroprotective [240]. 

Resveratrol has been found to be one of the strongest activators of SIRT-1 through an AMPK mediated mechanism. This is particularly important as SIRT-1 activation confers protection against aging-associated metabolic diseases, such as glucose metabolism impairment and carcinogenesis, apparently mimicking transcriptional aspects of dietary restriction [241]. In preclinical studies, resveratrol was found to protect against metabolic disease and weight gain in diet induced obesity models [242]. Moreover, its supplementation led to decreased adipogenesis and viability in preadipocytes and increased lipolysis and reduced lipogenesis in mature adipocytes [243].

Some studies conducted in both obese and non-obese subjects supplemented with resveratrol 75–2000 mg daily did not detect any metabolic effect [244,245]. Conversely, a cross-over study conducted on obese men taking 150 mg resveratrol daily for 30 days modestly mimicked the physiological effects of energy restriction as previously reported in rodent models [239]. A recent systematic review and metanalysis of 36 randomized controlled trials (RCTs) investigating the effect of resveratrol supplementation on weight loss showed that it significantly reduced weight, BMI, waist circumference, and fat mass with a significant increase in lean mass, but with no effect on leptin and adiponectin levels. Notably, despite the statistical significance, reductions were usually very small and not necessarily clinically relevant.

Altogether, studies supporting the use of Resveratrol as an anti-obesity drug are of low-quality, and the clinical relevance of its effects is uncertain. It could be however taken into consideration when associated to other weight loss interventions, given the strong preclinical evidence, the virtual absence of reported side effects and its small effect in weight reduction.

#### 3.4.6. Conjugated Linoleic Acid

Conjugated linoleic acid (CLA) is a group of isomers of linoleic Acid, polyunsaturated fatty acids that are found naturally in the meat and dairy products of ruminant animals. Cis-9, trans-11 CLA and trans-10, cis-12 CLA are some of the main active isomers [246]. The CLA content of food products is highly dependent on various factors, such as the type of feed [247,248], species and age of the animal [249], the rumen pH [250] and the time of the year [251].

CLA has been reported to exert anti-carcinogenic effects, improve body composition, and aid weight loss [252], modulate immune and/or inflammatory responses [253] while reducing cardiovascular risk [254,255]. CLA is GRAS (generally recognized as safe) in the United States since 2008 [252], with no major side effects reported in several studies except for the appearance of occasional GI complaints [256].

Several possible mechanisms have been suggested possibly aiding weight loss in animal models and human subjects. First, supplementation with CLA was shown to decrease the size of adipocytes, alter adipocyte differentiation, stimulate apoptotic pathways, and regulate lipid metabolism [257]. Moreover, some works suggested greater activation of PPAR-γ receptors and pro-inflammatory cytokines [246,253], fatty acids oxidation [258], and the browning of white adipose tissue as a mechanism of fat mobilization [259,260]. Finally, adding CLA to the diet could also alter the gut microbiota composition and associated gut metabolites [261,262], but more studies are needed to show that these changes play a role in weight loss. 

CLA studies in humans are difficult to interpret because of small sample sizes, variable doses, and isomers of CLA, a wide range of supplementation duration, and study population characteristics. A 2018 metanalysis evaluating the effect of CLA reported that CLA 3.4 g/day or more, for a minimum of 12 weeks, in subjects over 44 years of age had the greatest effect on body weight [263]. However, it should be highlighted that CLA supplementation resulted in only 1.3 kg reduction in body weight, of hardly any clinical relevance [263]. A recent review has highlighted that CLA, and primarily the 10,12 CLA isomer, consistently gives some degree of adiposity loss with different impact on different species, regardless of the presence of early symptoms of metabolic syndrome, without noticeable lean mass waste [264]. The same authors also report many studies failing to show any effects of mixed CLA supplementation on body parameters, and they propose several explanations such as the importance of a continuous dosing strategy for human efficacy, the need of higher doses of the active isomer of CLA, and differences in the basal metabolic rate between animals and humans [264].

In conclusion, the literature to date suggests that CLA, and primarily the 10,12 CLA isomer, promotes weight and fat loss in human subjects. Although its effectiveness seems to be clinically limited and the quality of evidence low, CLA could be considered as a treatment of obesity in addition to a dietary program given the strong preclinical evidence, the minor weight loss effect, and the very good safety profile.

#### 3.4.7. Aloe Vera

Aloe is a plant that belongs to the Liliaceae family; the most popular species is *Aloe Vera*, which has been used for centuries as a medicinal herb. Among its many applications, skin burns, and wound healing are the most common ones [265]. Various extracts of *A. vera*, used in animal and human studies, have also shown positive effects on glucose and lipid metabolism, gut microbiota, and blood pressure [266].

If its anti-obesity properties have been extensively demonstrated in animal models, only few human studies showed positive results regarding weight reduction [266]. In rodent models, *A. Vera* has been shown to reduce visceral fat accumulation, suppress lipogenesis related genes [267], enhance the expression of UCP-2 [268] and activate the AMPK pathway, leading to the acceleration of glucose and lipid oxidation in muscle and white adipose tissue [267]. 

In human studies, A. Vera supplementation was able to decrease serum triglycerides, total cholesterol, LDL, and fasting blood glucose level, as well as reducing HbA1c and insulin levels [265]. These effects on glucose metabolism are thought to be ascribable to acemannan, a mucopolysaccharide contained in A. Vera leaves, which is degraded by the intestinal microbiota to form oligosaccharides capable of inhibiting intestinal glucose absorption [269]; other properties of this plant, seen in animal studies, that may contribute to its positive effect on the lipid and glucose profile are represented by its ability to reduce oxidative stress, activate PPAR transcription, stimulate hepatic lipoprotein activity, and finally also activate hormone-sensitive lipase [266]. Furthermore, many active compounds present in this plant, such as acemannan and polyphenols, may increase the gut microbiota SCFAs production and stimulate gut anorexigenic hormones release, with the effect of reducing food intake and inducing weight loss [265]. 

In a 2013 randomized placebo-controlled trial, 136 obese prediabetic and early non-treated diabetic patients, were given 588 mg of *A. Vera* gel per day. At the end of the study, the aloe group achieved greater weight loss compared to placebo, showed a significant increase in lean body mass, a significant decrease in body fat mass and lower fasting blood glucose levels. The daily caloric intake, calculated from 24-h food recalls, decreased at four weeks in both groups and increased to baseline levels at week 8, suggesting an effect on weight loss independent of caloric intake. As the authors stated, the use of 24h food recalls to estimate daily caloric intake has several limitations and the estimation of body composition through bioimpedance analysis does not represent the gold standard, all these limitations possibly hindering the results observed [270].

A. vera has been shown to exert positive effects on glucose and lipid metabolism in both diabetic and nondiabetic patients. Evidence supporting its ability to induce weight loss, however, is limited and of low quality, so its consumption cannot be recommended for this purpose. Further studies are needed to confirm the results seen in animal studies and to elucidate the mechanisms supporting weight loss in humans.

#### 3.4.8. Flaxseed

Flaxseed is a functional food sourced from the flax plant; it is rich in α-linolenic acid (ALA), dietary fiber and lignans (phytoestrogen) [271]. Flaxseed contains approximately 22% ALA while flaxseed oil contains 50–62% ALA [272]. Flax and flaxseed have been historically used as a wound-healing and diuretic agent, pain, and cough reliever and to improve skin elasticity and its moisture holding capacity. Nowadays it is mostly used in cloths fabrication, animal feeding and as a functional food ingredient in juices, dairy, bakery, and meat products [273].

Results from reviews and metanalyses show that flaxseed consumption has beneficial effects on blood pressure [274], lipid profile [275] and glucose metabolism [276]. Evidence regarding its effect on body composition indices, however, is controversial [277]. Many flaxseed components may have positive effects on weight management: lignans are reported to reduce visceral fat and increase fat oxidation and adiponectin levels in mice [278], but their supplementation alone was not shown to be effective in improving body composition in humans [277]; soluble fiber, which represent up to 27% of flaxseed weight, may induce a feeling of fullness, delay gastric emptying and increase SCFA concentration in the gut, inducing satiety and promoting weight loss via GI hormones release [279]. Furthermore, ALA has been shown to increase adipose leptin expression in animal models [280] and flaxseed polysaccharides have been able to induce satiety improving leptin resistance together with enhancing lipolysis and suppressing lipogenesis through the AMPK signaling pathway [281]. Lastly, ALA metabolism products, eicosapentaenoic acid and docosahexaenoic acid, were able to induce adipocytes apoptosis, suppress appetite and enhance fat oxidation and energy expenditure in animal models, while human studies confirmed its potential benefit only in combination with exercise and hypocaloric diets [282]. A 2017 review and metanalysis including 45 RCTs, highlighted the effect of flaxseed supplementation on body weight and body composition [277]. Interestingly, only whole flaxseed was able to reduce body weight, BMI, and waist circumference, while flaxseed oil and lignans extracts did not show any benefit. Furthermore, a significant weight loss was observed only in subjects with BMI >27, eating more than 30 gr of flaxseed per day and in trials longer than 12 weeks. These findings suggest that the high fiber content or other compounds present in whole flaxseed are responsible for the higher weight loss. These results may also indicate that flaxseed supplementation has a cumulative and time dependent effect, probably because ALA, lignans or other flaxseed components build up their concentration in tissues over time or because of gut microbiota adaptations happening over a longer period. Among the studies analyzed, only few studies incorporated lifestyle advice or provided hypocaloric diets, and no data were reported regarding daily caloric intake, making it difficult to understand the mechanisms behind the weight reduction. Furthermore, the high heterogeneity of the studies regarding study populations (healthy subjects and subjects with different comorbidities) may hinder the results strength.

Overall, flaxseed showed promising weight reduction properties, backed by numerous health benefits, although the quality of the evidence supporting its application for this purpose is still low; however, considering the absence of side effects, its consumption may be considered for the treatment of obesity. To further understand the mechanisms promoting weight loss, studies analyzing appetite parameters alongside changes in daily caloric intake and gut hormones levels over time are needed. 

#### 3.4.9. Grapefruit

Grapefruit is a citrus fruit, known for its bitter taste. It is rich in water, vitamins, minerals, and polyphenols, such as phenolic acids, flavonoids, lignans and stilbenes [283]. Among its polyphenols, naringin and hesperidin have demonstrated antioxidant, lipid lowering and antihypertensive properties in animal models as well as human clinical trials [284]. Naringin and hesperidin are also able to improve glycemic control, enhancing insulin secretion and inhibiting gluconeogenesis [285]; like many other polyphenols, naringin has also been reported to stimulate the AMPK pathway, thus enhancing fatty acid oxidation and inhibiting lipogenesis [286]. Cells and animal studies demonstrated lipolytic effects of grapefruit extract and polyphenols through the inhibition of cAMP-phosphodiesterase and activation of hepatic peroxisome proliferator-activated receptor γ and α [287,288,289].

A 2014 review and metanalysis, taking into account RCTs that analyzed the effect of grapefruit on weight and body composition in humans, showed no benefit regarding weight loss, but a positive effect on waist circumference and body fat percentage reduction [283]. Of these, only one study reported a significant body weight reduction in 24 obese subjects eating half grapefruit before meals three times per day for 12 weeks, while the group assigned to receive grapefruit juice and grapefruit capsule did not achieve a significant weight loss [283]. Interestingly, none of the studies included in this literature revision reported a significant reduction in daily caloric intake when subjects following ad libitum diets or hypocaloric diets received grapefruit or grapefruit juice, compared to placebo. These results may indicate that grapefruit effect on adiposity is independent of caloric intake and may be a consequence of the whole fruit consumption. However, the paucity of trials analyzed in the revision and their short duration may limit the results. It should also be highlighted that different results may be seen if grapefruit is consumed in different quantities and modalities, taking into account that the included studies only investigated grapefruit or grapefruit juice as a preload before meals.

Overall, low-quality evidence supports grapefruit or grapefruit juice as a weight loss agent. Moreover, grapefruit may alter the metabolism of several drugs through cytochrome P450 interactions [283]. This aspect should be therefore be kept in mind in those on a daily pharmacological treatment, and the consumption of high amounts of grapefruit should be therefore discouraged in these subjects. Altogether, its use cannot be recommended at this time.

### 3.5. Carbohydrate Metabolism Improvement as Purported Mechanisms of Action

As a matter of fact, T2D and insulin resistance are two of the most important complications of obesity. A derangement in glucose metabolism in the obese patient usually has its roots in a vicious circle that includes the onset and progression of NAFLD. Food supplements that exert beneficial effects on glucose metabolism often do so through an improvement in the liver and increased expression of GLUT channels. It is therefore impossible to strictly classify supplements as lipid metabolism and carbohydrate metabolism improving. Here, we provide more detail on the food supplements proven to be more effective on glucose metabolism and insulin resistance improvement rather than in fat mass reduction (Table 5).

#### 3.5.1. Mangosteen

*Garcinia mangostana* L., commonly known as mangosteen, is a widespread evergreen tree in Southeast Asian countries, and its fruits have found many applications in traditional medicine for centuries. The main compounds are mangostins and isoprenylated xanthones, exerting antioxidant effects [290]. Recent evidence has also reported a possible role in the treatment of obesity and its comorbidities. 

In vitro studies have shown that alpha-mangostin acts as a strong inhibitor of pancreatic lipase, not different from the weight loss medication orlistat [291]. This compound was also reported to induce apoptosis of preadipocytes and enhance lipolysis through the inhibition of fatty acid synthase [292]. In rodent models, mangosteen supplementation led to a glucose lowering effect that could likely be due to hyperplasia of pancreatic beta cells and alpha glucosidase activity [293,294,295]. Moreover, alpha-mangostin treated diet induced obesity mice experienced weight loss, improved glucose and lipid profile and reduced liver fat accumulation through a Peroxisome proliferator-activated receptor gamma and SIRT-1-AMPK pathway [296,297]. Four to sixteen week-long studies conducted on human subjects and assessing the effect of 200–400 mg of mangosteen extracts reported significant weight loss and waist circumference reduction, and an excellent safety and tolerability profile [298,299,300,301]. In our hands, a 26-week-long mangosteen extract supplementation led to glucose metabolism improvement in insulin resistant female subjects with obesity, with a frank decrease of HOMA-IR, independent of body weight change [302].

Considering the promising but scanty and low-quality evidence, mangosteen should not be encouraged nor recommended against as a treatment of obesity and its complications such as insulin resistance, also given its optimal cost-to-benefit ratio.

#### 3.5.2. Chromium

Chromium (Cr) is an essential nutrient widely distributed in the human diet. The main food sources of Cr are meat, nuts, cereal grains, molasses, and brewer’s yeast. The exact mechanism of Cr is not well understood, however, salt forms, such as Cr nicotinate, Cr chloride, and Cr picolinate, are believed to be associated with an increase in the activity of insulin [303]. Cr may also contribute to weight loss by suppressing appetite and by stimulating human thermogenesis, through sensitization of insulin-sensitive glucoreceptors in the brain, thus increasing energy expenditure [304].

A recent metanalysis evaluated the efficacy of oral Cr supplementation from randomized controlled trials published from 1996 to 2017 [305]. Of all selected trials, twelve included subjects who were overweight or obese. Different forms of Cr were used in the included trials, with most administering Cr picolinate, followed by Cr nicotinate and Cr-enriched yeast. The present metanalysis indicates that Cr supplementation for less than 13 weeks is associated with significant overall placebo-controlled weight loss in individuals who were overweight and obese, in terms of BMI and body fat percentage. These findings are partially in contrast with a previous metanalysis by Onakpoya et al., suggesting that Cr should be supplemented for at least 16 weeks in order to reach a maximal weight loss of 1 Kg [306]. Notably, in three included RCTs Cr was administered in association with other compounds, making its individual effect indistinguishable [307,308]. Subgroup analysis by dosage of Cr supplementation showed significant improvement in the mean change of body weight in trials which administered a dose of Cr ≤ 400 μg/dl. Even though this metanalysis did not outline any evidence of specific adverse effects with Cr supplementation, previous studies have reported concerns regarding the safety of Cr picolinate supplementation which could be responsible for renal and hepatic impairment [309]. Nevertheless, several studies reported no significant body weight loss after Cr supplementation compared to placebo or even reported significant weight gain. However, it should be noted that in one case no dietary control was performed, possibly hindering results, and in the other subjects were not selected based on BMI, and lean patients may as well have been enrolled [310,311]. Pointing in the same direction, a systematic review investigating weight loss and metabolic and hormonal variables in patients with polycystic ovary syndrome suggested that Cr supplementation has no beneficial effects [312].

In conclusion, the evidence supporting the use of Cr for weight loss is moderate, although clinical relevance remains uncertain, possibly because of the presence of different forms of supplementation; further investigation through larger studies is necessary. However, given the less favorable safety profile compared to other food supplements, its use cannot be recommended nor discouraged at his time.

#### 3.5.3. Lipoic Acid

Alpha-lipoic acid (LA), or 1,2-dithiolan-3-pentanoic acid is enzymatically synthesized in the mitochondria from octanoic acid. In addition to the synthesis, LA is also absorbed intact from food sources and accumulates transiently in many tissues. LA has been described as a powerful biological antioxidant with glucose lowering effects; it is used to improve cardiovascular, cognitive, and neuromuscular deficits related to aging and is able to modulate various pathways of inflammation [313,314,315]. The typical food sources of LA are meat, offal, and to a lesser extent, fruit and vegetables [316]. Given the limited quantities present in these nutritional sources, LA does not seem to be consumed in an appreciable way in the western diet. Rather, dietary supplements that typically range from 50 to 600 mg are the primary sources of LA and most of the information on its bioavailability comes from studies using supplements.

LA seems to lead to increased GLUT4 expression on the cell membrane of skeletal muscle and adipocyte cells, through a PI3K-dependent mechanism that involves the insulin signaling cascade [317]. Intraperitoneal administration of LA on Zucker rats led to a skeletal muscle increase in glucose uptake in acute (100 mg/kg body weight for 1 h) and in chronic conditions (50 mg/kg bodyweight for 10 days) [318]. Combining LA supplementation (30 mg/kg per day for 15 days) with physical exercise in an animal model of insulin resistance, additive effects of improved glucose tolerance and intracellular glucose transport have been observed. A potential mechanism for this additive effect is the upregulation of GLUT4 protein expression in muscle combined with the enhanced translocation of GLUT4 on the LA-induced plasma membrane [319,320]. Improvements in glucose metabolism have also been observed in humans with T2D treated with intravenous or oral LA [321,322]. A systematic review including ten double blind, placebo-controlled RCTs that investigated LA for weight loss purposes showed that its supplementation was associated with a statistically significant 1.27 kg greater mean weight loss compared to placebo [323].

Several human studies have assessed the efficacy and safety of LA as a food supplement. Clinical trials ALADIN (I, II and III), SYDNEY (I and II), and ORPIL used LA supplements of up to 2400 mg/day with no adverse effects reported compared to placebo. Moreover, oral supplementation of 1800 mg LA for 6 months did not cause significant adverse effects compared to placebo [324]. LA has also been associated with cases of insulin autoimmune syndrome (IAS). Also known as Hirata disease, IAS is a rare genetic disease that has occurred most often in Japanese subjects. IAS patients develop antibodies to insulin, causing episodes of hypoglycaemia [325]. 

Altogether, LA seems to have a clinically relevant glucose lowering effect, but evidence supporting its use towards weight loss are still of low-quality. Its supplementation may be considered for the treatment of obesity, especially when T2D coexists, under the supervision of experienced health professionals, given the possible side effects that have been reported.

## 4. Conclusions

Given the present literature revision, it is possible to conclude that many of the presented food supplements are likely to exert an anti-obesogenic effect in the absence of significant adverse events. However, none of this is capable of inducing a clinically relevant weight loss, with the most effective ones leading to a mere 2-kg reduction. Given the current coronavirus disease 19 (COVID-19) pandemic, and with obesity being a well-established risk factor for worse prognosis [326,327,328], it is now of the utmost importance to find safe and effective ways to tackle weight excess.

Supported by current evidence, we propose a possible combination leveraging all mechanisms of action that could pave the way for future studies investigating the weight loss effect and safety profile of such product. 

**Figure 1:**
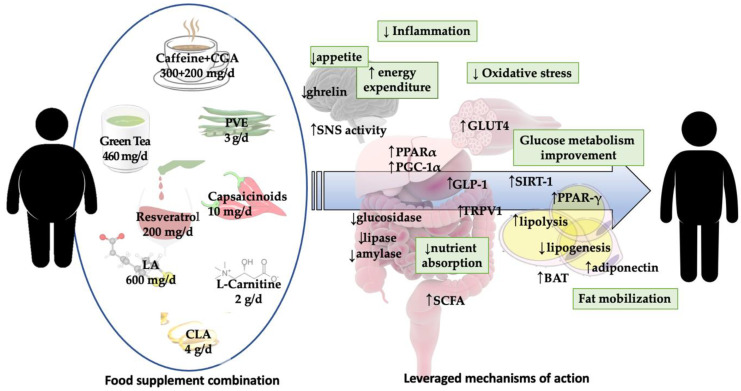
Proposed food supplement combination leveraging multiple mechanisms of action to aid weight loss and metabolism improvement based on the current state of the art. Green tea was shown to inhibit pancreatic lipase, amylase, and glucosidase in the gastrointestinal tract reducing the absorption of nutrients and leading to the presence of undigested carbohydrates in the GI tract, in turn driving the microbiota to produce short-chain fatty acids (SCFA). Through an AMPK dependent mechanism, it also inhibits lipogenesis and induces lipolysis. Phaseolus vulgaris extract (PVE) contains phaseolin, an α-amylase inhibitor whose function impairs the absorption of carbohydrates. Caffeine suppresses hunger and stimulates energy expenditure through increased excitability of the sympathetic nervous system (SNS), increased fat oxidation and Brown Adipose Tissue (BAT) activation. Capsaicinoids activate the Transient Receptor Potential Channel Vanilloid type-1 (TRPV1) leading to Glucagon like peptide 1 (GLP-1) release, increased fat oxidation, increased Sirtuin-1 (SIRT-1) expression. They also suppress ghrelin release and increase adiponectin, PPARα and PGC-1α expression. They finally regulate gluconeogenesis and glycogen synthesis genes improving insulin resistance. L-Carnitine was shown to improve insulin resistance, increase acetyl-coenzyme A and glucose supply to the brain leading to increased energy expenditure; it facilitates activated long chain fatty acids transportation into mitochondria, playing an important role in β-oxidation. It also modulates lipid metabolism. Resveratrol increases SIRT-1 expression, decreases adipogenesis and viability in maturing preadipocytes and modulates lipid metabolism in mature adipocytes. Conjugated linoleic acid (CLA) decreases the size of adipocytes, alters adipocyte differentiation, regulates lipid metabolism and activates of PPAR-γ receptors. Lipoic acid increases GLUT4 expression on the cell membrane of skeletal muscle and adipocyte cells leading to increased glucose uptake, hence improved glucose tolerance, chlorogenic acid (CGA).

To inhibit the absorption of nutrients, we suggest using phaseolus vulgaris extract (PVE) at a dosage of 3000 mg (1000 mg per meal) daily, and green tea derived epigallocatechin (EGCG) at a dosage of 500 mg daily. In order to reduce appetite and possibly increase energy expenditure, we propose the use of coffee derived caffeine (300 mg/daily) and chrologenic acid (200 mg/daily). Chili pepper derived capsaicinoids or capsinoids may also be considered, at a dosage of 10 mg and 3 mg, respectively, together with L-C at a dosage of 2 g daily, that can also increase fat mobilization. Similarly enhancing beta oxidation and inhibiting lipogenesis, resveratrol and CLA may be considered, given their proven efficacy and absence of reported adverse events, at a dosage of 200 mg and 4 g daily, respectively. Finally, carbohydrate metabolism may be improved with glucose lowering lipoic acid at a dosage of 600 mg daily, with its long-standing history in the treatment of T2D. Most of the cited dietary supplements were also proven to exert anti-inflammatory and antioxidant effects, possibly aiding the resolution of low-grade chronic inflammation typical of weight excess and metabolic derangements (Figure 1).

A specifically designed, placebo-controlled study investigating the proposed combination for weight loss purposes is now needed, in order to confirm the safety profile, the absence of detrimental interactions between the suggested compounds, and the presence of an additive or synergistic effect possibly aiding weight loss in a safe and effective way.

## Figures and Tables

**Table 1:** Food supplements with a primary impact on nutrients absorption.

Food Supplement	Dosage	Mechanisms of Action	Quality of Evidence	Side Effects	Recommendation
**Green Tea**	100–460 mg/day	lipase, amylase, glucosidase inhibition *#; gut microbiota modification *#	moderate	None	A
**Ginseng**	100 mg–18 g/day	lipase inhibition *#; appetite hormonwhie levels modification *	low	None	B
**White Kidney Bean**	1–3 g/day	α-amylase inhibition *#; antioxidant *, anticarcinogenic *#, anti-inflammatory*#, glucose lowering *#, and cardioprotective properties *#	moderate	Lectin’s toxicity (abstent in phaseolus vulgaris extracts)	A
**Chitosan**	1–4.5 g/day	absorption of dietary fats inhibition *#; decreased lipid peroxidation; adipogenesis inhibition *	low	Gastrointestinal discomfort and bloating	B
**β-Glucans**	5–9 g/day	lipid and carbohydrate absorption inhibition *#; satiety induction *#; PYY-NPY axis activation *#	low	None	B
**Psyllium**	3–10.5 g/day	carbohydrate absorption inhibition *#; decreased serum lipids *#; delayed gastric emptying *#; glucose control *#; satiety induction *#	low	Gastrointestinal discomfort and bloating	B
**Glucomannan**	2–3 g/day	delayed gastric emptying *#, increased satiety *#; decreased post-prandial glucose concentration	low	None	B
**Guar Gum**	9–30 g/day	increased postprandial fullness #, appetite and food intake reduction *#	low	Gastrointestinal discomfort, bloating, diarrhea	B
**Agar**	180 g/day	delayed gastric emptying #; increased satiety *#	low	None	B
**Inulin**	8–30 g/day	carbohydrate absorption inhibition *#; gut microbiota modulation *#; increased satiety *#	low	None	B

* denotes preclinical evidence, # denotes clinical evidence, A is for possibly recommended, B is for undetermined recommendation.

**Table 2:** Food supplements with a primary impact on appetite regulation.

Food Supplement	Dosage	Mechanisms of Action	Quality of Evidence	Side Effects	Recommendation
Caralluma	1 g/day	fatty acid synthesis inhibition and enhanced fatty acid oxidation *; ghrelin/neuropeptide Y expression inhibition *#	low	None	B
Spirulina	1–4.5 g/day	decreased appetite *#; adipogenesis inhibition *; browning induction *	low	None	B
Whey protein	∼100–600 g/week	increased satiety hormones; decreased appetite; enhanced fat mass oxidation; enhanced lean mass preservation *#	moderate	None	A
Coffee, caffeine and chlorogenic acids	60 mg–1000 g/day	hunger suppression; energy expenditure stimulation; increased fat oxidation and brown adipose tissue activation *#	moderate	None	A
Bitter orange	10–400 mg/day	suppressed appetite *#; energy expenditure and lipolysis increase *#	low	None	B
Guarana	240–285 mg/day	decreased appetite #; increased energy expenditure and fat oxidation *#; adipogenesis inhibition *; browning induction *	low	Gastrointestinal discomfort; insomnia, migraine, tachycardia	B

* denotes preclinical evidence, # denotes clinical evidence, A is for possibly recommended, B is for undetermined recommendation.

**Table 3:** Food supplements with a primary impact on energy expenditure modulation.

Food Supplement	Dosage	Mechanisms of Action	Quality of Evidence	Recommendation	Side Effects
Capsaicin, capsaicinoids and capsinoids	10–30 mg/day	browning, thermogenesis, fat oxidation *#, energy expenditure induction #	low	B	Gastrointestinal discomfort and diarrhea
Curcumin	70 mg–12 g/day	adipogenesis inhibition *; insulin-sensitizing and anti-inflammatory properties *#	low	B	Gastrointestinal discomfort, headache, urticaria
L-Carnitine	10 mg–4 g/day	increased energy expenditure and fat oxidation *#; improved insulin resistance *#; modulation of regulators of lipid catabolism or adipogenesis *; induction of satiety *#	low	B	None

* denotes preclinical evidence, # denotes clinical evidence, B is for undetermined recommendation.

**Table 4:** Food supplements with a primary impact on fat metabolism.

Food Supplement	Dosage	Mechanisms of Action	Quality of Evidence	Side Effects	Recommendation
Pyruvate	5–44 g/day	reduced insulin level and increased acetylCoA concentrations *#	low	None	B
Dyacilglycerol	1.1–1.2 g/day	enhanced fat oxidation *#; reduced postprandial triglycerides *#	moderate	None	A
Licorice	300–900 mg/day	reduced serum lipids *#; improved hepatic steatosis through beta-oxidation induction *#	low	Increased blood pressure, hypernatremia	C
Garcinia Gambogia	400–2400 mg/day	decreased lipogenesis and increased lipolysis *	low	Hepatotoxicity, diarrhea	C
Resveratrol	75–2000 mg/day	decreased adipogenesis; increased lipolysis;reduced lipogenesis *#	low	None	A
Conjugated linoleic acid	1.5–6.8 g/day	decreased adipocytes size; inhibited adipogenesis; reducted lipogenesis; induced browining *; gut microbiota modification *#;	low	Occasional gastrointestinal discomfort	A
Aloe vera	588–700 mg/day	Improved glucose and lipid metabolism *#; reduced oxidative stress * ; inhibited lipogenesis *	low	None	B
Flaxseed	20–50 g/day	increased saxiety *# and lipolysis *; inhibited lipogenesis *	low	None	A
Grapefruit	81–142 mg/day	improved glycemic control, enhanced insulin secretion and inhibited gluconeogenesis *#; increased fat oxidation and reduced lipogenesis *	low	Possible alteration of several drugs metabolism	B

* denotes preclinical evidence, # denotes clinical evidence, A is for possibly recommended, B is for undetermined recommendation, C is for not recommended.

**Table 5:** Food supplements with a primary impact on carbohydrate metabolism.

Food Supplement	Dosage	Mechanisms of Action	Quality of Evidence	Side Effects	Recommendation
Mangosteen	200–400 mg/day	inhibition of pancreatic lipase and fatty acid synthase *; improved glucose metabolism *#	Low	None	B
Chromium	157–1000 μg/day	Energy expenditure increase *#; appetite suppression *#; improved glucose metabolism *#	Moderate	Diarrhea, vertigo, headache, urticaria	B
Lipoic Acid	300–2400 mg/day	improved glucose metabolism *#; appetite suppression *#; increase of lipolysis and reduction of lipogenesis *	Low	Gastrointestinal disconfort, urticaria, hypoglycemia	B

* denotes preclinical evidence, # denotes clinical evidence, B is for undetermined recommendation, C is for not recommended.
